# Practices and challenges of family involvement in neonatal intensive care units (NICUs) across Asia: a multinational survey of the Asian neonatal network

**DOI:** 10.3389/fped.2026.1753007

**Published:** 2026-04-30

**Authors:** Alicia May Lim, Bin Huey Quek, Zubair Amin, Woei Bing Poon, Su Jin Cho, Young-Ah Youn, Seok Chiong Chee, Siew Hong Neoh, Chatchay Prempunpong, Pathoporn Prempraphan, Ming-Chih Lin, Hsiang Yu Lin, Ma Lourdes S. Imperial, Rinawati Rohsiswatmo, Dewi Rizalya, Yuri Ozawa, Satoshi Kusuda, Miyake Fuyu, Tetsuya Isayama

**Affiliations:** 1 Department of Neonatology, KK Women’s and Children’s Hospital, Singapore, Singapore; 2 Department of Neonatology, Khoo Teck Puat- National University Children’s Medical Institute, National University Hospital, Singapore, Singapore; 3 Department of Neonatology and Developmental Medicine, Singapore General Hospital, Singapore, Singapore; 4 Department of Pediatrics, College of Medicine, Ewha Womans University, Seoul, Republic of Korea; 5 Department of Pediatrics, College of Medicine, The Catholic University of Korea, Seoul, Republic of Korea; 6 School of Medicine, Faculty of Health and Medical Sciences, Taylor’s University, Subang Jaya, Selangor, Malaysia; 7 Department of Pediatrics, Faculty of Medicine, Ramathibodi Hospital, Mahidol University, Bangkok, Thailand; 8 Department of Pediatrics, Faculty of Medicine, Vajira Hospital, Navamindradhiraj University, Bangkok, Thailand; 9 Department of Post-Baccalaureate Medicine, College of Medicine, National Chung Hsing University, Taichung, Taiwan; 10 China Medical University Children’s Hospital, Taichung, Taiwan; 11 Department of Newborn Medicine, Dr. Jose Fabella Memorial Hospital, Manila, Philippines; 12 Department of Paediatrics, Department of Child Health, Faculty of Medicine Universitas Indonesia, Cipto Mangunkusumo General Hospital, Jakarta, Indonesia; 13 Budhi Mulia Mother and Child Hospital, Pekanbaru, Indonesia; 14 Department of Pediatrics, Kyorin University School of Medicine, Mitaka, Tokyo, Japan; 15 Division of Neonatology, National Center for Child Health and Development, Setagaya, Tokyo, Japan; 16 Neonatal Research Network of Japan, Shinjuku, Tokyo, Japan

**Keywords:** family integrated care, kangaroo care, kangaroo mother care, neonatal intensive care unit, neonate

## Abstract

**Objective:**

This study aimed to explore the role of family involvement in neonatal intensive care units (NICUs) across Southeast and East Asia, focusing on the implementation of family-integrated care, kangaroo mother care, and developmental care practices. The study sought to identify variations in care practices between high-income and low- and middle-income countries and to provide actionable insights for improving neonatal outcomes.

**Design:**

A multinational survey was conducted across 336 NICUs in eight Asian neonatal networks (Japan, Indonesia, Malaysia, the Philippines, Singapore, South Korea, Taiwan, and Thailand) as part of the AsianNeo Network in 2021. The survey, adapted from the International Network for Evaluation of Outcomes (iNeo) tool, assessed family-centred care practices, kangaroo mother care, and developmental care.

**Results:**

Significant differences were observed between high-income and low- and middle-income countries. High-income countries had better facilities for parental involvement, such as family rooms and extended visitation policies, but were more restrictive in kangaroo mother care duration. Low- and middle-income countries prioritized maternal involvement, with longer kangaroo mother care durations and greater emphasis on breastfeeding support. Developmental care practices, such as cyclical light exposure and noise reduction, were more prevalent in high-income countries.

**Conclusions:**

The study highlights the need for context-specific strategies to enhance family-centred care in NICUs. Low- and middle-income countries should invest in infrastructure and training to support family-integrated care and developmental practices, while high-income countries can adopt more flexible kangaroo mother care policies. Collaborative efforts within networks like AsianNeo can facilitate knowledge exchange and improve neonatal care outcomes across diverse resource settings.

## 1 Introduction

Admission to the neonatal intensive care unit (NICU) immediately after birth disrupts the critical process of parent-infant bonding, potentially resulting in long-term consequences such as parental well-being, infant development, and the parent-infant relationship. These disruptions often persist beyond the neonatal period, adversely affecting cognitive and socioemotional outcomes in children (1). In response to these challenges, contemporary NICU care models have evolved to prioritize family-centred interventions, including family integrated care, kangaroo mother care, and developmental care practices.

Family integrated care, a multifaceted and evidence-based model, empowers parents to assume the role of primary caregivers within the NICU. By fostering collaborative partnerships between parents and healthcare teams, family integrated care directly addresses the adverse effects of NICU stay, mitigating parent-infant separation and promoting sustained parent-infant interactions. This approach integrates psychoeducational, communicative, and environmental strategies to equip parents with the emotional, cognitive, and practical skills necessary to care for their infants both during and beyond the NICU stay. Evidence underscores family integrated care's benefits, including enhanced infant feeding outcomes, increased breastfeeding rates at discharge, accelerated infant growth, and improved parental well-being (2, 3). Importantly, family integrated care serves as a critical mediator for long-term neurodevelopmental and behavioural improvements in infants, positioning it as a cornerstone of modern neonatal care (2, 3). Successful implementation of family integrated care, however, hinges on multidisciplinary commitment, staff training, and a cultural shift toward shared decision-making, wherein healthcare providers transition from traditional authority roles to collaborative facilitators. Despite its demonstrated efficacy, resource disparities across NICUs—ranging from staffing capacity to institutional priorities—pose challenges to consistent, full-scale adoption of family integrated care (2).

Kangaroo mother care is an evidence-based care package that involves placing the newborn in an upright, chest-to-chest position on a parent's bare torso, secured with a supportive wrap, to maintain continuous skin-to-skin contact. Unlike standard intermittent skin-to-skin holding, kangaroo mother care emphasizes prolonged duration and is paired with exclusive breastfeeding to optimize outcomes. Recognized by the World Health Organization as a natural, cost-effective intervention, kangaroo mother care is recommended as a global standard of care for all infants—transcending geographic, economic, or healthcare disparities. Studies demonstrate that kangaroo mother care reduces neonatal mortality, stabilizes physiological parameters (e.g., temperature, heart rate), enhances neurodevelopmental trajectories, prolongs breastfeeding success, and strengthens parent-infant bonding. Despite its proven benefits and endorsement from the World Health Organization, implementation remains inconsistent and underprioritized in clinical settings (4–6).

Developmental care represents a holistic, evidence-based framework designed to minimize stressors and support the neurodevelopmental needs of infants in the NICU. This approach prioritizes individualized strategies such as minimizing disruptive external stimuli (e.g., dimmed lighting for visual sensitivity, noise reduction for auditory overload, and gentle handling to regulate tactile or vestibular input), clustering medical procedures to reduce disturbances, and employing containment techniques like swaddling to mimic the nurturing womb environment (7). These practices are frequently systematized through programmes such as the Newborn Individualized Developmental Care and Assessment Program, which customizes caregiving to an infant's behavioural cues. By aligning medical care with developmental trajectory, this model aims to foster neuroprotective conditions, thereby promoting optimal brain maturation, accelerating weight gain, shortening hospitalization periods, and reducing physiological instability linked to chronic stress. Collectively, these interventions not only address immediate clinical outcomes but also strive to improve long-term health trajectories for preterm infants (8, 9).

The implementation of family integrated care, kangaroo mother care, and developmental care is marked by significant variability and inconsistency across countries, regions, and resource settings, underscoring a persistent gap between evidence-based recommendations and clinical practice (10, 11). Understanding the impact of resource limitations and other barriers on the practical application of these interconnected care models is essential for NICUs to develop targeted strategies to enhance their integration into neonatal care. Despite the growing recognition of the importance of family-centered care, there remains a notable lack of multinational research, particularly in Southeast Asia and East Asia, on the implementation of family integrated care and related practices. To address this gap, we conducted a comprehensive survey among NICUs in Southeast Asia and East Asia to explore the role of families in neonatal care and to identify variations in practices between higher- and lower-resourced settings. Our study aims to illuminate the existing disparities in care delivery and provide actionable insights to promote the widespread adoption of these evidence-based practices, ultimately improving outcomes for neonates and their families.

## 2 Methods

### 2.1 Study setting and network

This study was conducted as part of the AsianNeo Network, a collaborative initiative established to provide an international platform for paediatricians, neonatologists, researchers, and other healthcare providers. The primary aim of AsianNeo is to enhance the understanding of health systems, clinical management, and outcomes for sick newborn infants across Asian countries (12). By fostering collaboration and knowledge exchange, the network seeks to improve the quality of neonatal care. At the time of this survey, AsianNeo comprised eight neonatal networks with 446 NICUs:, Indonesia (38), Japan (225), Malaysia (35), Philippines (16), Singapore (3), South Korea (13), Taiwan (23), and Thailand (93). These countries represent diverse income levels, wealth equality, and baseline perinatal indices, offering a unique opportunity to explore variations in neonatal care practices. Through this network, member countries share best practices and research findings, enabling mutual learning and improvement in neonatal care delivery (12, 13).

### 2.2 Survey development and administration

The survey questionnaire was adapted from a previously developed tool by the International Network for Evaluation of Outcomes (iNeo), with permission granted by the iNeo group for its use and modifications (13, 14). The original survey was a comprehensive survey that explored clinical management, care practice, and resources available to NICUs. In this paper, we report findings of family integrated care, kangaroo mother care and developmental care.

The survey was conducted in English and distributed using the Survey Monkey platform. To accommodate non-English speaking countries, the questionnaire was translated into local languages, allowing participants to respond in their native language. The survey was distributed to participating NICUs through representatives of each neonatal network within the AsianNeo Network. Only one representative, usually the head of the unit or clinical director or a designee, from each NICU responded on behalf of the unit. Participants were instructed to provide responses based on their unit's protocols and practices as of 2021, the year the survey was administered, rather than their personal preferences.

### 2.3 Ethical considerations

The study protocol was approved by the Research Ethics Board of the National Center for Child Health and Development, Tokyo, Japan (ID 2020-244). Written informed consent was obtained from all neonatologists who participated in the survey. Participation was voluntary, and the survey responses were anonymized to ensure confidentiality.

### 2.4 Data analysis

Descriptive statistics were used to summarize the characteristics of the NICUs. Continuous variables were expressed as medians with interquartile ranges, while categorical data were presented as absolute frequencies and percentages. To explore variations in care practices, responses were further stratified and compared between high-income countries and low- and middle-income countries, as classified by the Organization for Economic Co-operation and Development (15). We classified Japan, Singapore, South Korea and Taiwan and as high-income countries; while, Indonesia, Malaysia, Philippines, and Thailand were classified as low- and middle-income countries. Chi-square tests was used to analyze the differences between the high-income and middle/low-income countries. Statistical significance was set at *p* < 0.05. All analyses were performed using Stata 17 (StataCorp. 2021. College Station, TX: StataCorp LLC). In order to maintain the integrity of data, the incomplete survey was excluded from analysis.

## 3 Results

The overall response rate was 75% (337/446). 1 questionnaire was incomplete and had to be excluded. The majority (69%) of respondents worked in units which provided both level 2 and level 3 care. Forty percent of the units were nationally funded.

### 3.1 Comparison of facilities between high-income and middle/low-income countries

Table 1 shows NICU resources between high-income and low- and middle-income countries. Supplementary Appendix S1 provides more detailed information by country.

**Table 1:** Demographics of participants and resources in the NICU when comparing high-income and middle/low income countries.

Clinical characteristics	*N* (%)	High income (*n* = 185)	Low/middle income (*n* = 151)	*P*—Value
Type of NICU,				
Level 3	96/314 (30)	73/173 (42)	23/141 (16)	<0.01
Level 3 and Level 2	216/314 (69)	98/173 (57)	118/141 (84)	<0.01
Level 2	2/336 (0.6)	2/173 (1)	0 (0)	0.5
Family rooms for overnight stay	97/287 (34)	42/147 (29)	55/140 (39)	0.05
Joint rooms for family and infants (Level 3/NICU)	24/287 (8)	13/147 (9)	11/140 (8)	0.7
Joint rooms for family and infants (Special care/Level 2)	38/286 (13)	16/146 (11)	22/140 (16)	0.2
Rooms for parents to stay with their babies for trial run before taking baby home	168/287 (59)	87/147 (59)	81/140 (58)	<0.01
Parent's lounge	74/287 (26)	31/147 (21)	43/140 (30)	0.06
Parent kitchen/cooking facility	15/287 (5)	6/147 (4)	9/140 (6)	0.4
Parent rest room (with beds/possibility to lie down)	72/287 (25)	22/147 (15)	50/140 (36)	<0.01
Breast milk pumping rooms				
I. Yes	232/285 (81)	112/147 (76)	120/140 (85)	0.05
Visitation by parents				
I. Anytime including rounds and handover	124/280 (44)	58/147 (31)	66/140 (47)	0.08
II. Limited hours (≥8 h per day)	42/280 (15)	23/147 (12)	19/140 (14)	0.88
III. Limited hours (<8 h per day)	89/280 (32)	50/147 (27)	39/140 (28)	0.81
IV. Limited days	18/280 (6)	14/147 (8)	4/140 (3)	0.13
V. Not allowed to visit but can see infants through the glass windows	7/280 (3)	1 (0.6)	6/140 (4)	0.09

Data are presented as *n*/*N* (%), where *n* is the number of institutions that selected the given response, *N* is the total number of institutions that provided a valid response (excluding those that answered “Cannot answer”), and % indicates the corresponding percentage.

Significant differences were observed in the availability of family rooms for overnight stays between high-income countries and low- and middle-income countries. While a higher proportion of high-income countries reported having rooms available for both parents, low- and middle-income countries were significantly more likely to provide rooms exclusively for mothers [34% (48/140) in low- and middle-income countries vs. 3% (4/147) in high-income countries, *p* < 0.01]. A similar trend was observed in Level 2 NICUs, where low- and middle-income countries had a higher availability of mother-only rooms compared to high-income countries [16% (22/140) vs. 3% (4/147) respectively, *p* < 0.01]. This trend extended to other family-centered facilities, such as parent lounges and rest rooms, where low- and middle-income countries were more likely to provide spaces specifically for mothers.

Breast milk expression rooms were more widely available in low- and middle-income countries compared to high-income countries (79% vs. 61%, *p* = 0.05). Regarding visitation policies, the most common practice across NICUs was limited visitation hours (less than 8 h per day). However, high-income countries were more likely to permit visitation by extended family members, including grandparents, siblings, and parents' friends, compared to low- and middle-income countries.

### 3.2 Kangaroo mother care

Table 2 highlights the differences in kangaroo mother care and skin-to-skin practices between among NICUs from high-income countries and low- and middle-income countries. A detailed country-level breakdown is provided in Supplementary Appendix S2.

**Table 2:** Kangaroo mother care differences between high-income and middle/low-income countries.

Kangaroo mother care	*N* (%)	High income (*n* = 185)	Low/Middle income (*n* = 151)	*P*-Value
Do you allow infants with each of the following conditions to receive kangaroo mother care or skin-to-skin if they are stable otherwise and their mother want to do it?				
I. Infants on CPAP	238/287 (83)	128/147 (87)	114/140 (81)	0.20
II. Infants on mechanical ventilation	92/287 (32)	60/147 (41)	32/140 (23)	0.02
III. Infants with PICC	238/287 (83)	130/147 (88)	108/140 (77)	0.81
Once infants are stable, how many hours per day of kangaroo mother care or skin-to-skin is allowed?				
I. 24 h per day/7 days	16/283 (5)	3/145 (2)	13/138 (9)	<0.01
II. 12–23 h per day	8/283 (2)	0/145 (0)	8/138 (6)	<0.01
III. 6–11 h per day	22/283 (7)	2/145 (1)	20/138 (14)	<0.01
IV. 2–5 h per day	62/283 (18)	20/145 (14)	42/138 (30)	<0.01
V. <2 h per day	160/283 (48)	114/145 (79)	46/138 (33)	<0.01
VI. No kangaroo mother care/skin-to-skin at all	10/283 (3)	5/145 (3)	5/138 (4)	0.76
Once infants become stable, how many days per week kangaroo mother care or skin-to-skin are allowed?				
I. Everyday	168/281 (60)	63/144 (43)	105/137 (76)	<0.01
II. None	15/281 (5)	7/144 (5)	8/137 (6)	0.50

Data are presented as *n*/*N* (%), where *n* is the number of institutions that selected the given response, *N* is the total number of institutions that provided a valid response (excluding those that answered “Cannot answer”), and % indicates the corresponding percentage.

CPAP, continuous positive airway pressure; PICC, peripherally inserted central catheter.

In high-income countries, a higher proportion of NICUs permitted kangaroo mother care or skin-to-skin care for neonates receiving mechanical ventilation compared to low- and middle-income countries [41% (60/147) in high-income countries vs. 23% (32/140) in low- and middle-income countries, *p* = 0.02]. However, there were no significant differences between high-income countries and low- and middle-income countries in allowing kangaroo mother care for neonates on CPAP or those with a peripherally inserted central catheter.

Overall, NICUs in low- and middle-income countries reported allowing longer durations of kangaroo mother care or skin-to-skin compared to those in high-income countries. Among high-income countries, 79% (114/145) of NICUs allowed less than 2 h of kangaroo mother care or skin-to-skin time, whereas only 33% (46/138) of NICUs in low- and middle-income countries reported such restrictions (*p* < 0.01). Additionally, once infants were stabilized, NICUs in low- and middle-income countries were more likely to permit kangaroo mother care or skin-to-skin daily compared to those in high-income countries [76% (105/137) vs. 43% (63/144), *p* < 0.01].

### 3.3 Developmental care

Table 3 outlines the differences in developmental care practices between high-income countries and low- and middle-income countries. A detailed country-level breakdown is provided in Supplementary Appendix S3. Significant differences were observed between high-income countries and low- and middle-income countries in the provision of developmental care. High-income countries reported a higher prevalence of cyclical light exposure [81% (119/147) vs. 52% (73/140), *p* < 0.01] and developmentally supportive interventions [64% (94/147) vs. 38% (51/136), *p* < 0.01] compared to low- and middle-income countries.

**Table 3:** Developmental care.

Developmental care	*N* (%)	High income (*n* = 185)	Low/Middle income (*n* = 151)	*P*-Value
Exposure to cyclical day—Light on and off (yes)	192/287 (69)	119/147 (81)	73/140 (52)	<0.01
Reduction of noise levels (yes)	165/286 (58)	94/147 (64)	71/139 (51)	0.49
Developmentally supportive interventions such as NIDCAP (yes)	145/283 (51)	94/147 (64)	51/136 (38)	<0.01
Formal parental teaching including breastfeeding (yes)	251/287 (87)	132/147 (90)	119/140 (85)	0.12
Training parents for infant CPR (yes)	157/284 (55)	87/144 (60)	70/140 (50)	0.92

Data are presented as *n*/*N* (%), where *n* is the number of institutions that selected the given response, N is the total number of institutions that provided a valid response (excluding those that answered “Cannot answer”), and % indicates the corresponding percentage.

NIDCAP, newborn individualized developmental care and assessment program; CPR, cardiopulmonary resuscitation.

## 4 Discussion

This study, first of its kind from Southeast and East Asia, provides a comprehensive comparison of neonatal care practices, including family-centred care, kangaroo mother care, and developmental care, between high-income countries and low- and middle-income countries within the AsianNeo Network. The findings highlight significant differences in resource availability, care practices, and family involvement, reflecting the influence of socioeconomic factors on neonatal care delivery.

### 4.1 Family integrated care and facility resources

Our results reveal notable differences in the availability of family-centred facilities between high-income countries and low- and middle-income countries. While high-income countries were more likely to provide rooms for both parents to stay prior to discharge, low- and middle-income countries predominantly offered facilities exclusively for mothers. This disparity may reflect cultural norms and resource constraints in low- and middle-income countries, where maternal involvement is often prioritized due to limited infrastructure. However, the higher availability of breast milk expression rooms in low- and middle-income countries suggests a strong emphasis on supporting breastfeeding, which is a cornerstone of neonatal care in resource-limited settings. Conversely, the restricted visitation policies in low- and middle-income countries, particularly the limited hours and exclusion of extended family members, may reflect challenges in managing space and infection control in high-volume NICUs (16).

### 4.2 Kangaroo mother care and skin-to-skin practices

The study highlights contrasting approaches to kangaroo mother care and skin-to-skin practices between high-income countries and low- and middle-income countries. While high-income countries were more likely to permit kangaroo mother care or skin-to-skin for neonates on mechanical ventilation, low- and middle-income countries reported longer daily durations of kangaroo mother care or skin-to-skin and greater flexibility in allowing these practices once infants were stabilized. This suggests that low- and middle-income countries may prioritize kangaroo mother care as a low-cost, high-impact intervention to improve neonatal outcomes, particularly in settings with limited access to advanced medical technologies. The reluctance of high-income countries to extend kangaroo mother care or skin-to-skin time may reflect stricter adherence to clinical protocols or concerns about workload and staffing (6). These findings emphasize the need for high-income countries to adopt more flexible kangaroo mother care policies, particularly for stable infants, to maximize the benefits of this evidence-based practice.

### 4.3 Developmental care

Significant differences were observed in the provision of developmental care, with high-income countries reporting higher rates of cyclical light exposure and developmentally supportive interventions compared to low- and middle-income countries. These findings align with the greater availability of resources and specialized training in high-income countries, enabling the implementation of advanced developmental care practices. However, the lower prevalence of such interventions in low- and middle-income countries highlights a critical gap in neonatal care that may impact long-term neurodevelopmental outcomes. Efforts to promote developmental care in low- and middle-income countries should focus on cost-effective, scalable interventions, such as training healthcare providers in developmental support techniques and integrating developmental care into routine neonatal care protocols (10, 11).

Globally, neonatal mortality rates have declined over the past decades, yet significant disparities persist between high-income countries and low- and middle-income countries, as well as between rural and urban regions (17–22). Barriers to optimal neonatal care include shortages of human and physical resources, inadequate infrastructure, lack of standardized guidelines, and poor communication (22, 23). Our study highlights that many NICUs, regardless of income level, lack adequate facilities to support optimal family involvement in care. Key gaps include insufficient parent lounges, rest rooms, and rooms for parents to stay with their newborns prior to discharge. Interestingly, low- and middle-income countries in this survey were more likely to provide facilities for mothers to stay with their infants compared to high-income countries, which may reflect cultural norms that emphasize maternal caregiving roles.

The involvement of fathers in NICU care, who traditionally received less attention regarding the availability of facilities, has gained increasing recognition. An integrative review by Holm et al. found that participation and collaboration are critical for fathers of preterm infants, who often experience high levels of stress and anxiety during NICU admissions (24). Fathers frequently report feeling excluded from care and must advocate for their own involvement (24, 25). A cohort study demonstrated that those participating in family-integrated care models, which included single-family rooms and shared caregiving responsibilities for the mother-infant dyad, experienced lower stress levels and greater involvement in their infant's care. This approach also reduced paternal depressive symptoms and enhanced parent-infant bonding (26).

A qualitative study on factors influencing family integrated care implementation identified that adequate facilities are essential for parents to feel welcomed and integrated into the NICU environment. These facilities should meet parents' daily needs, such as rest areas and private spaces. Additionally, policies supporting continuous parental presence and rooming-in are critical for successful family integrated care implementation (16). A systematic review on evidence-based design for neonatal units found that single-family rooms are ideal, offering benefits such as improved infant sleep, enhanced privacy, greater parental involvement, better infection control, and reduced noise levels. Infants in single-family rooms also experienced fewer apnoeic events, lower rates of nosocomial sepsis, and improved nutritional outcomes, including earlier transition to enteral feeding and greater weight gain at discharge. Long-term benefits included better attention, reduced hypertonicity, and lower psychological stress. Furthermore, single-family rooms were associated with shorter hospital stays, fewer readmissions, and higher parental satisfaction (27); however, this provision may not be feasible for many NICUs due to space and manpower constraints.

A systematic review and meta-analysis by Reiter et al. examined parental participation in neonatal care in low- and middle-income countries and found that it was associated with reduced mortality, increased breastfeeding rates, and decreased hospital readmissions. Although not statistically significant, improvements were also observed in infant growth, short-term neurodevelopment, and parental well-being (28). To enhance parental participation in family integrated care, high-income countries can learn from low- and middle-income countries by promoting 24 h parental presence, which fosters partnerships between parents and healthcare teams, enhances parents' sense of safety and control, and reduces anxiety (29). Conversely, low- and middle-income countries can focus on increasing paternal involvement, moving beyond the current emphasis on maternal caregiving, to promote shared parental responsibility and improve outcomes for preterm infants.

### 4.4 Strengths and limitations

This is the first study comparing practices of NICU resources for families, developmental care, and kangaroo mother care across Southeast and East Asia with a mix of high-income countries and low- and middle-income countries. It provides a comparison of neonatal care practices across diverse socioeconomic settings. However, the findings should be interpreted in light of certain limitations. The survey relied on self-reported data, which may be subject to reporting bias. As the survey participants were from various NICUs participating in the AsianNeo network, the reporting may not be reflective of national policy. A larger number of representatives from each unit could have provided better insight to unit practices. This was a survey with fixed responses, a more in-depth understanding of practices could have been more informative. Additionally, the study did not explore the impact of these practices on clinical outcomes, which should be addressed in future research.

## 5 Conclusions

The disparities identified in this study underscore the need for context-specific strategies to improve neonatal care across different resource settings. In low- and middle-income countries, investments in training in developmental care are essential to enhance developmental support while maintaining the strengths of existing practices, such as kangaroo mother care. In high-income countries, there is an opportunity to adopt more flexible and inclusive family integrated care policies, drawing on the successful practices observed in low- and middle-income countries. Collaborative initiatives within networks like AsianNeo can facilitate knowledge exchange and capacity building, enabling countries to learn from each other's experiences and adapt best practices to their unique contexts.

## Data Availability

The original contributions presented in the study are included in the article/Supplementary Material, further inquiries can be directed to the corresponding author.
