# Supplementary material for: Practices and challenges of family involvement in neonatal intensive care units (NICUs) across Asia: a multinational survey of the Asian neonatal network
Source: Front Pediatr. 2026 Apr 30;14:1753007. doi: 10.3389/fped.2026.1753007 (PMC13171482; doi:10.3389/fped.2026.1753007)
Supplement: Supplementary file 1 [file Datasheet1.pdf]

Appendix 1: Graphical representation of facilities by country

(*Low/middle income country, ** High income country)

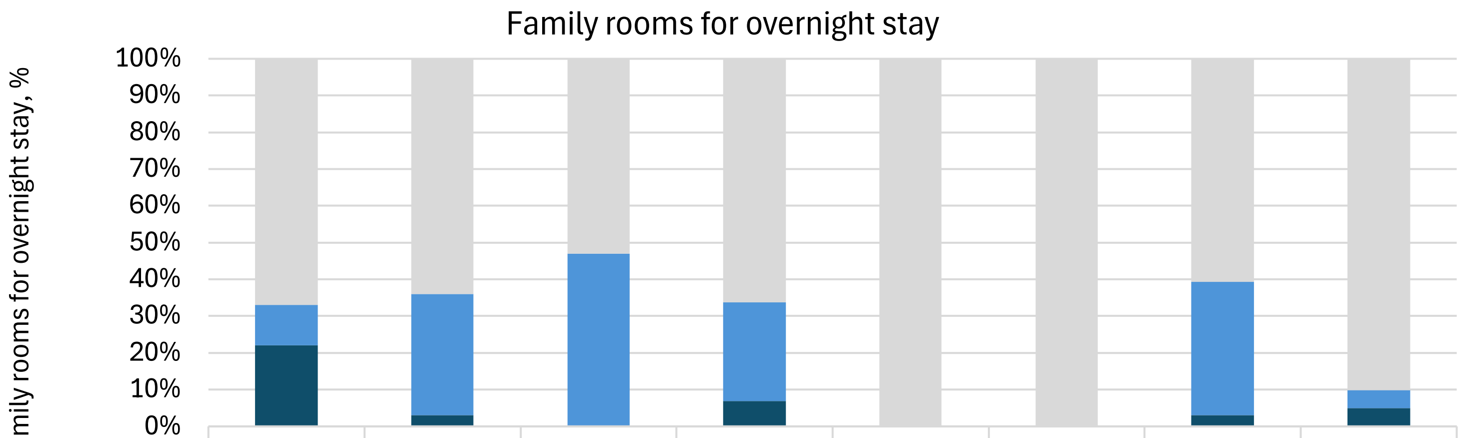

	Indonesia*	Japan**	Malaysia*	Philippines*	Singapore**	South Korea**	Thailand*	Taiwan**
None	67	64	53	67	100	100	60	91
Mothers Only	11	33	47	27	0	0	36	5
Both Parents	22	3	0	7	0	0	3	5

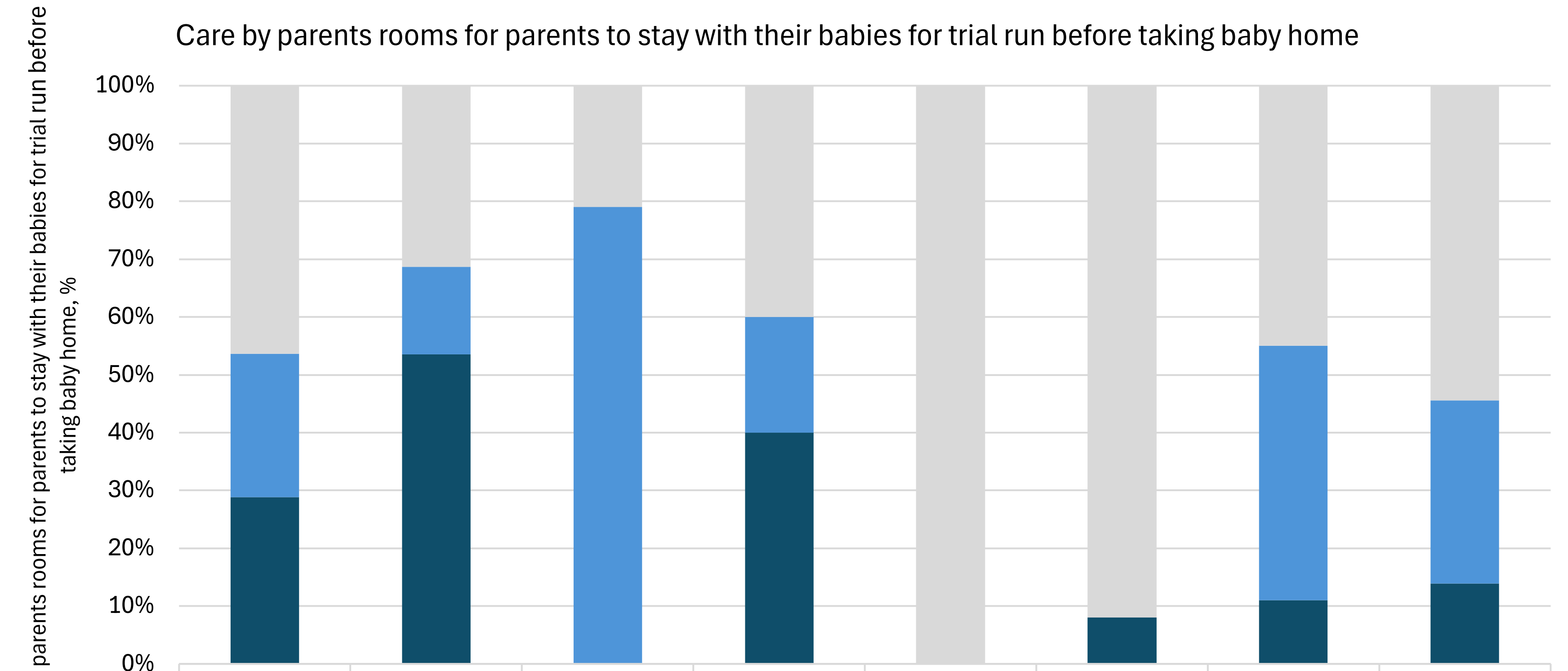

	Indonesia*	Japan**	Malaysia*	Philippines*	Singapore**	South Korea**	Thailand*	Taiwan**
None	58	31	21	40	100	92	45	55
Mothers Only	31	15	79	20	0	0	44	32
Both Parents	36	53	0	40	0	8	11	14

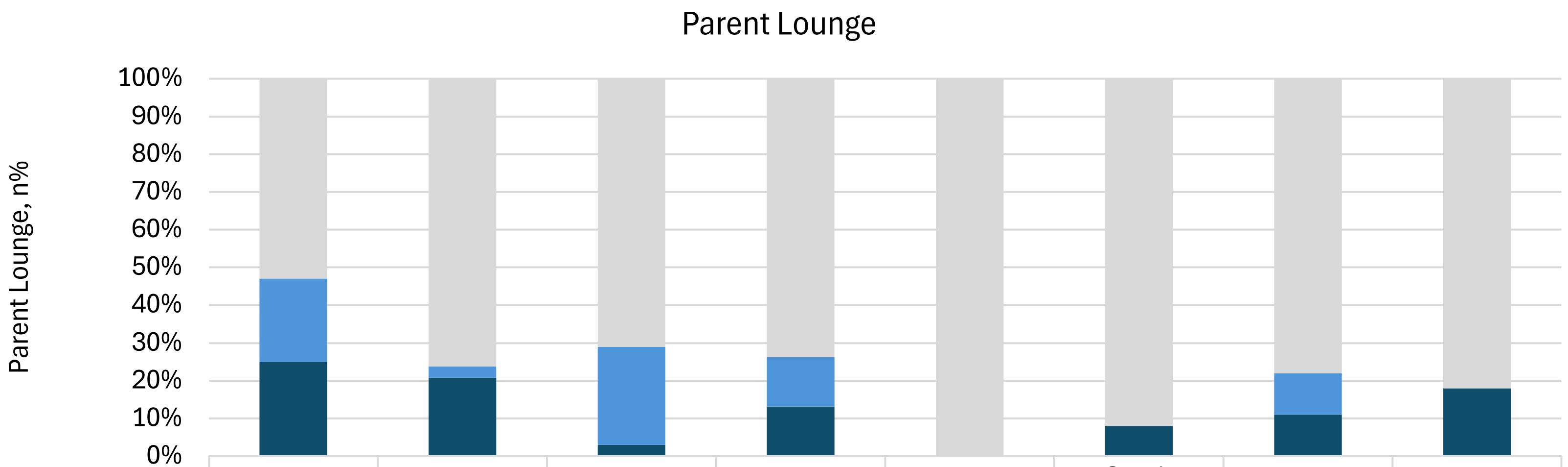

	Indonesia*	Japan**	Malaysia*	Philippines*	Singapore**	South Korea**	Thailand*	Taiwan**
Series4	0							
None	53	77	71	73	100	92	78	82
Mothers Only	22	3	26	13	0	0	11	0
Both Parents	25	21	3	13	0	8	11	18

Country

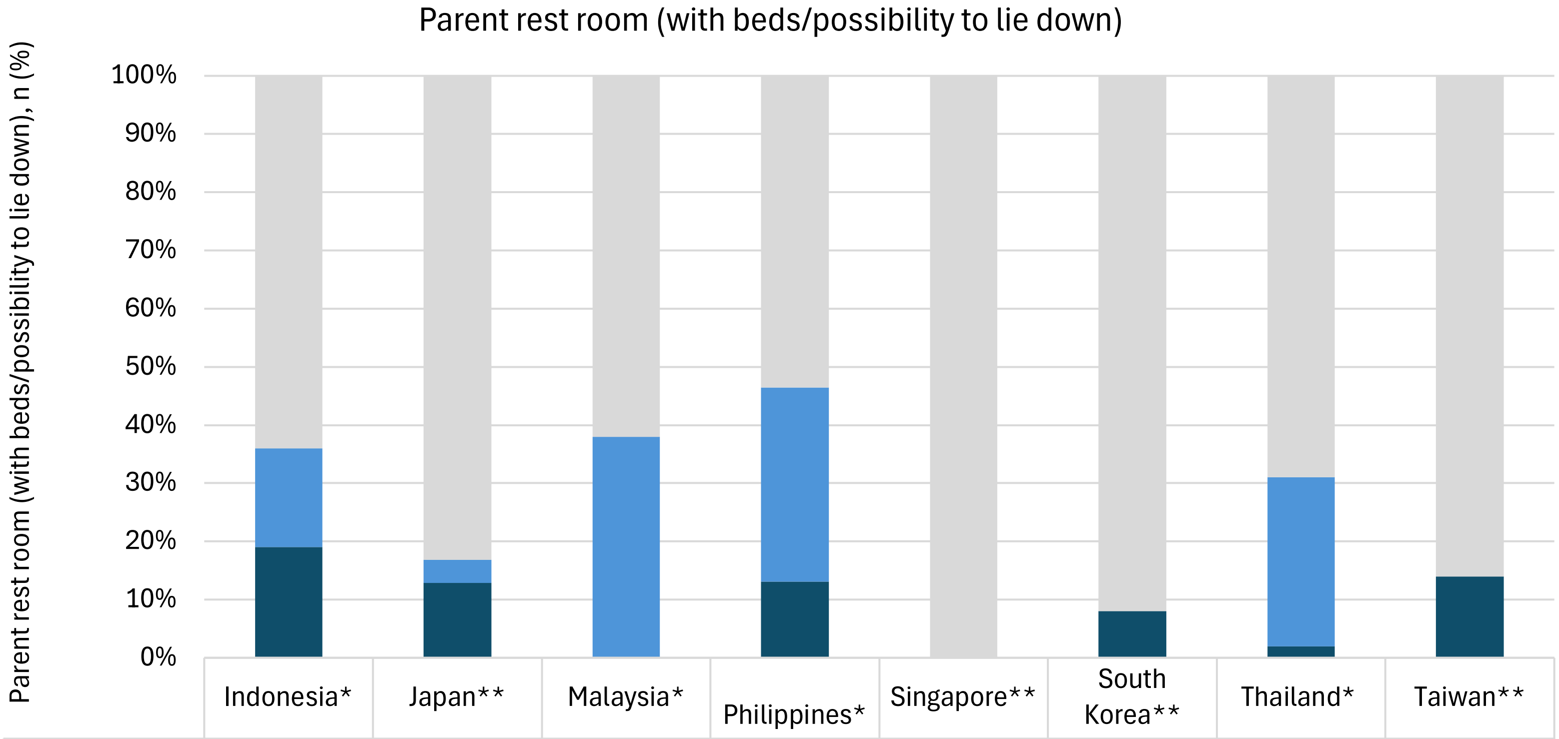

	Indonesia*	Japan**	Malaysia*	Philippines*	Singapore**	South Korea**	Thailand*	Taiwan**
None	64	84	62	53	100	92	69	86
Mothers Only	17	4	38	33	0	0	29	0
Both Parents	19	13	0	13	0	8	2	14

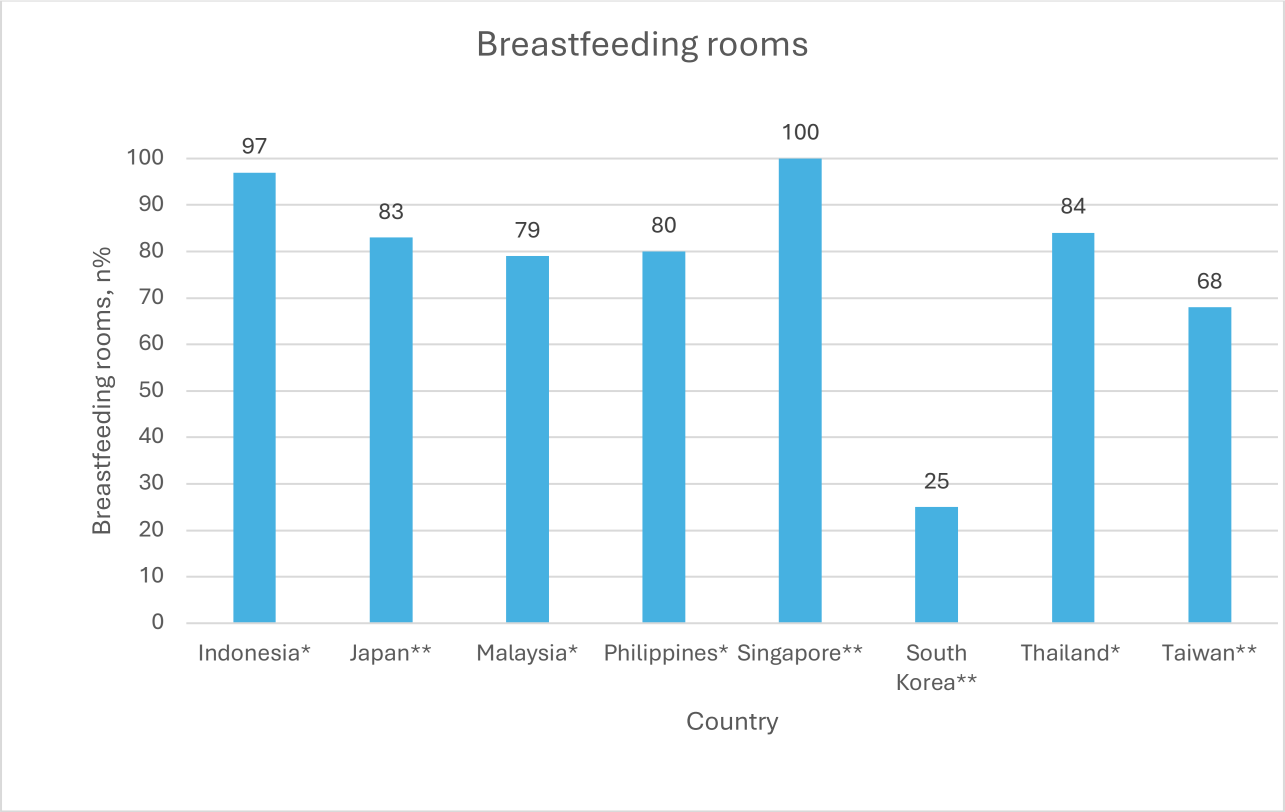
Breastfeeding rooms
Breastfeeding rooms, n%
Country
0
10
20
30
40
50
60
70
80
90
100
97
83
79
80
100
25
84
68
Indonesia*
Japan**
Malaysia*
Philippines*
Singapore**
South Korea**
Thailand*
Taiwan**

Appendix 2: Graphical Representation by country of developmental care

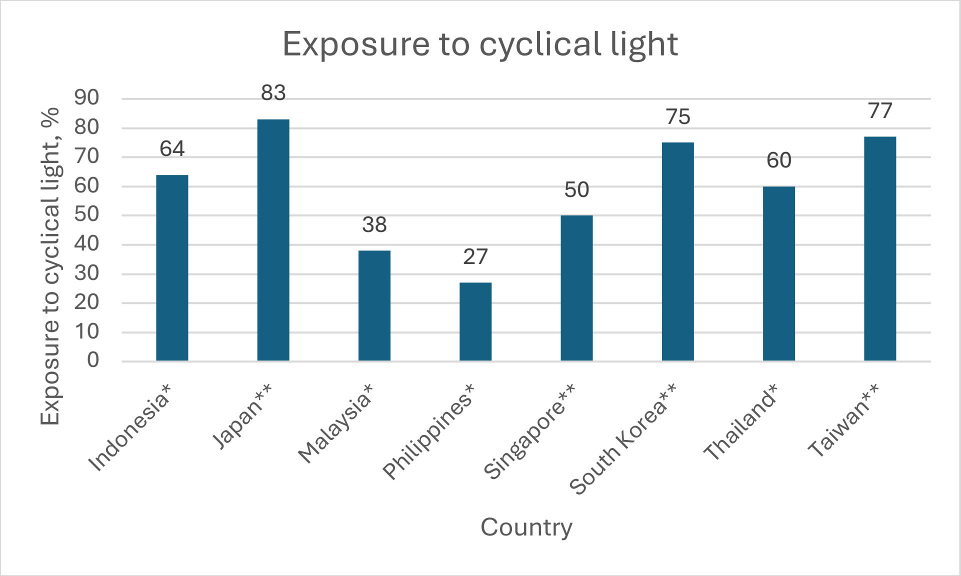

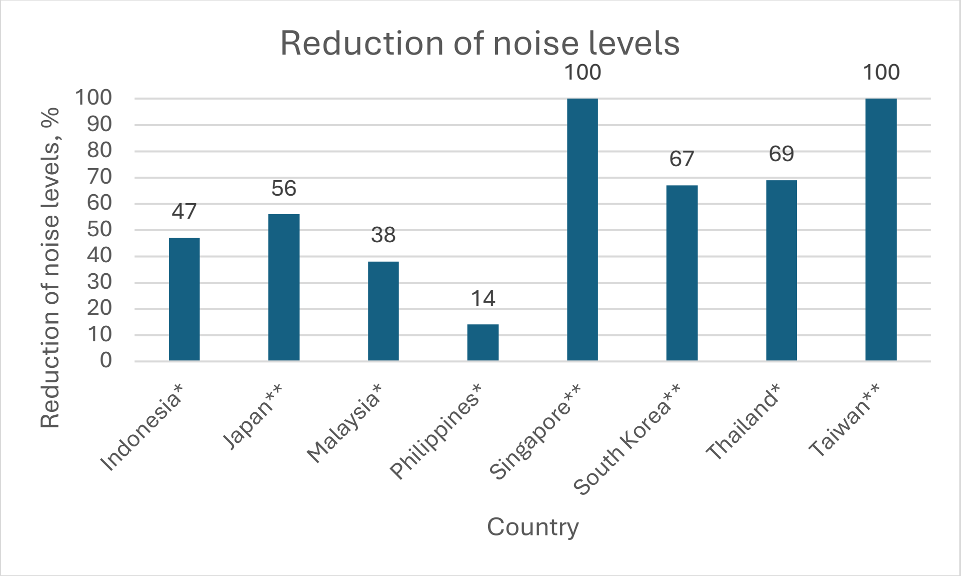
Reduction of noise levels
Reduction of noise levels, %
100
90
80
70
60
50
40
30
20
10
0
47
56
38
14
100
67
69
100
Indonesia*
Japan**
Malaysia*
Philippines*
Singapore**
South Korea**
Thailand*
Taiwan**
Country

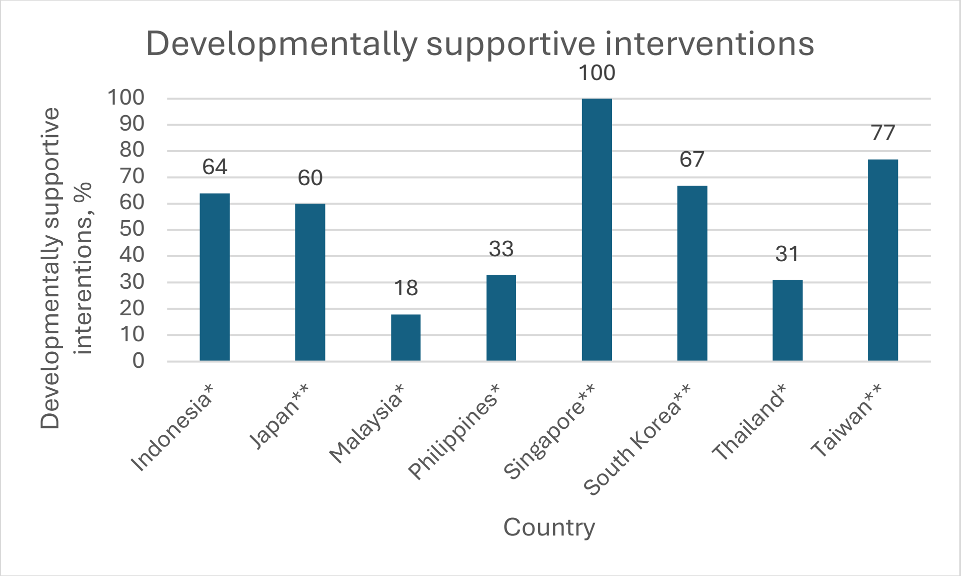
Developmentally supportive interventions
Developmentally supportive interentions, %
100
90
80
70
60
50
40
30
20
10
0
64
60
18
33
100
67
31
77
Indonesia*
Japan**
Malaysia*
Philippines*
Singapore**
South Korea**
Thailand*
Taiwan**
Country

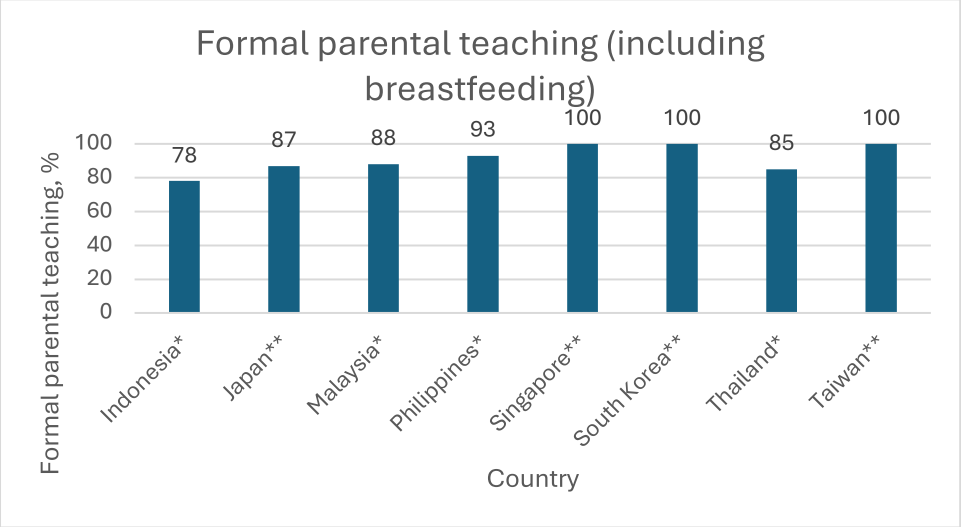
Formal parental teaching (including breastfeeding)
Formal parental teaching, %
100
80
60
40
20
0
78
87
88
93
100
100
85
100
Indonesia*
Japan**
Malaysia*
Philippines*
Singapore**
South Korea**
Thailand*
Taiwan**
Country

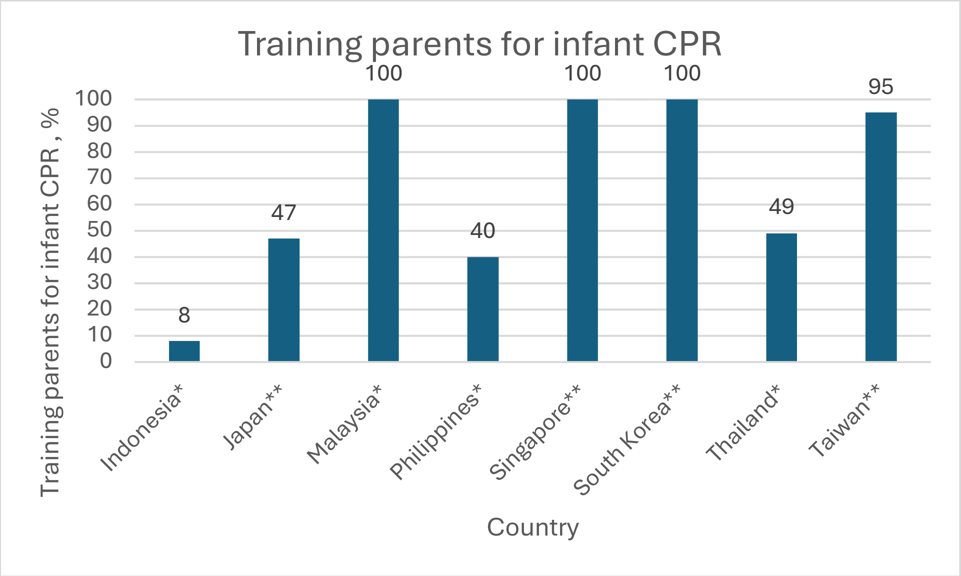
Training parents for infant CPR
Training parents for infant CPR , %
100
90
80
70
60
50
40
30
20
10
0
8
47
100
40
100
100
49
95
Indonesia*
Japan**
Malaysia*
Philippines*
Singapore**
South Korea**
Thailand*
Taiwan**
Country

Appendix 3:Graphical representation by country for Kangaroo Mother Care/Skin to Skin

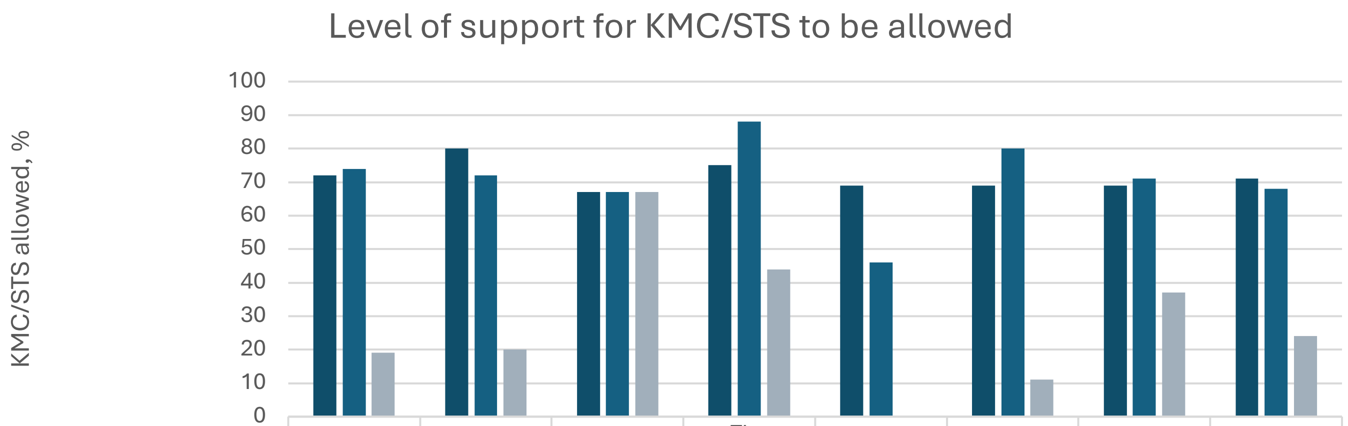

	Thailand	Taiwan	Singapore	The Philippines	Korea	Malaysia	Japan	Indonesia
PICC	72	80	67	75	69	69	69	71
CPAP	74	72	67	88	46	80	71	68
Mechanical Ventilation	19	20	67	44	0	11	37	24

Country

PICC CPAP Mechanical Ventilation

Duration of KMC/STS once infant is stabilized

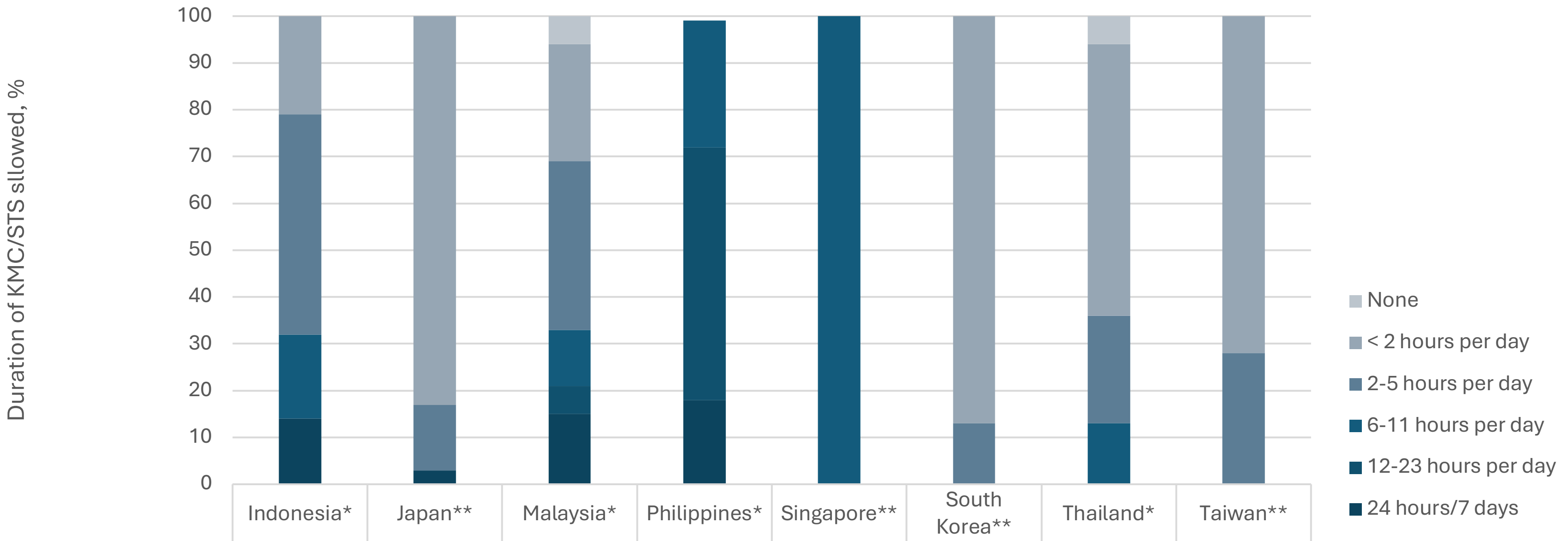

	Indonesia*	Japan**	Malaysia*	Philippines*	Singapore**	South Korea**	Thailand*	Taiwan**
None	0	0	6	0	0	0	6	0
< 2 hours per day	21	83	25	0	0	87	58	72
2-5 hours per day	47	14	36	0	0	13	23	28
6-11 hours per day	18	0	12	27	100	0	13	0
12-23 hours per day	0	0	6	54	0	0	0	0
24 hours/7 days	14	3	15	18	0	0	0	0

Country

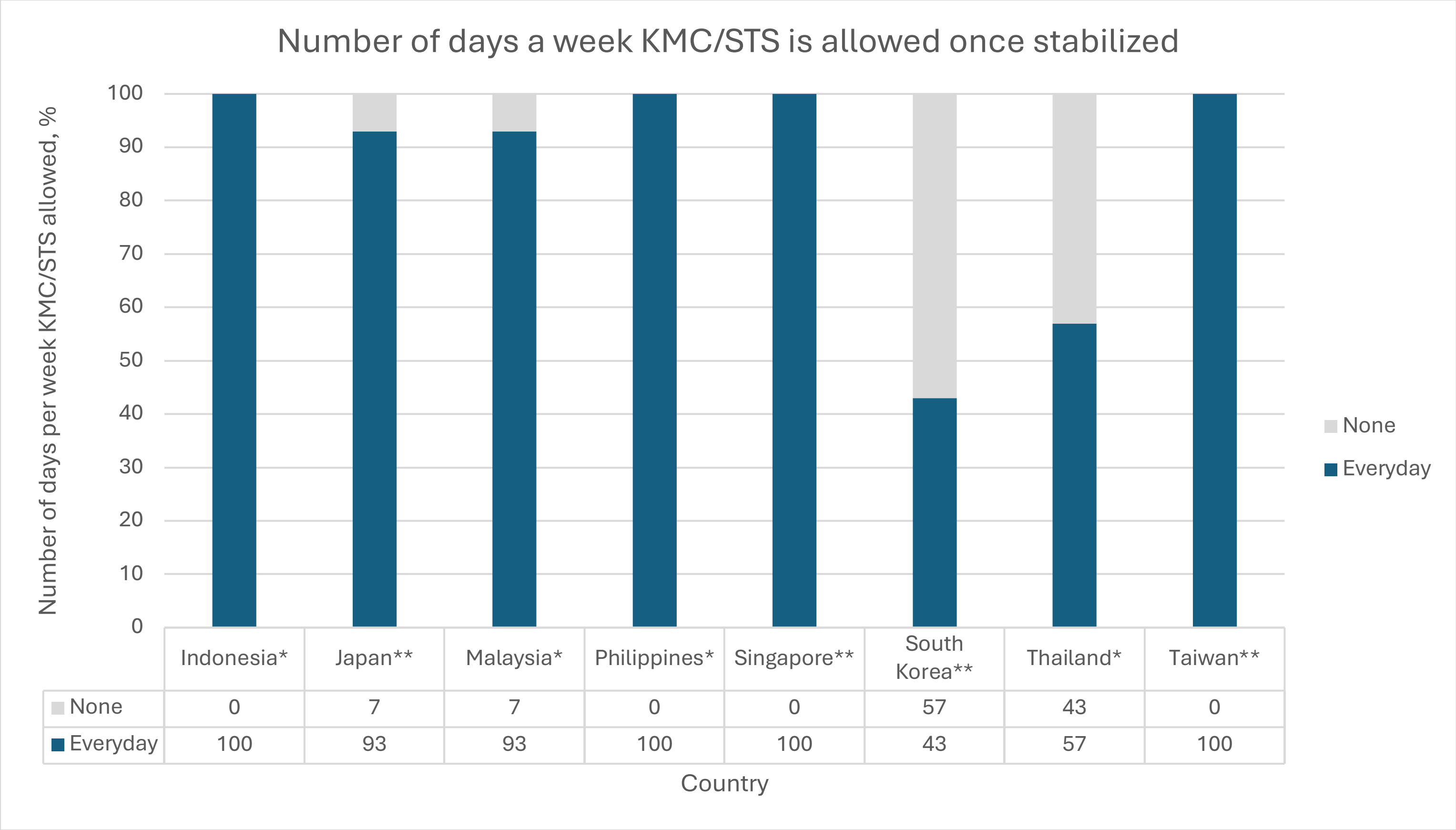

	Indonesia*	Japan**	Malaysia*	Philippines*	Singapore**	South Korea**	Thailand*	Taiwan**
None	0	7	7	0	0	57	43	0
Everyday	100	93	93	100	100	43	57	100
